# Diffusion-weighted imaging-based radiomics in epithelial ovarian tumors: Assessment of histologic subtype

**DOI:** 10.3389/fonc.2022.978123

**Published:** 2022-12-05

**Authors:** Yi Xu, Hong-Jian Luo, Jialiang Ren, Li-mei Guo, Jinliang Niu, Xiaoli Song

**Affiliations:** ^1^ Department of Radiology, Second Hospital of Shanxi Medical University, Taiyuan, Shanxi, China; ^2^ Department of Radiology, The Third Affiliated Hospital of Zunyi Medical University (The First People’s Hospital of Zunyi), Zunyi, Guizhou, China; ^3^ GE Healthcare, Beijing, China

**Keywords:** epithelial ovarian tumors, diffusion weighted imaging, apparent diffusion coefficient, radiomics, nomogram

## Abstract

**Background:**

Epithelial ovarian tumors (EOTs) are a group of heterogeneous neoplasms. It is importance to preoperatively differentiate the histologic subtypes of EOTs. Our study aims to investigate the potential of radiomics signatures based on diffusion-weighted imaging (DWI) and apparent diffusion coefficient (ADC) maps for categorizing EOTs.

**Methods:**

This retrospectively enrolled 146 EOTs patients [34 with borderline EOT(BEOT), 30 with type I and 82 with type II epithelial ovarian cancer (EOC)]. A total of 390 radiomics features were extracted from DWI and ADC maps. Subsequently, the LASSO algorithm was used to reduce the feature dimensions. A radiomics signature was established using multivariable logistic regression method with 3-fold cross-validation and repeated 50 times. Patients with bilateral lesions were included in the validation cohort and a heuristic selection method was established to select the tumor with maximum probability for final consideration. A nomogram incorporating the radiomics signature and clinical characteristics was also developed. Receiver operator characteristic, decision curve analysis (DCA), and net reclassification index (NRI) were applied to compare the diagnostic performance and clinical net benefit of predictive model.

**Results:**

For distinguishing BEOT from EOC, the radiomics signature and nomogram showed more favorable discrimination than the clinical model (0.915 vs. 0.852 and 0.954 vs. 0.852, respectively) in the training cohort. In classifying early-stage type I and type II EOC, the radiomics signature exhibited superior diagnostic performance over the clinical model (AUC 0.905 vs. 0.735). The diagnostic efficacy of the nomogram was the same as that of the radiomics model with NRI value of -0.1591 (*P* = 0.7268). DCA also showed that the radiomics model and combined model had higher net benefits than the clinical model.

**Conclusion:**

Radiomics analysis based on DWI, and ADC maps serve as an effective quantitative approach to categorize EOTs.

## Introduction

Epithelial ovarian tumors (EOTs) are a group of heterogeneous neoplasms and are subdivided into three subtypes: benign, borderline, and malignant tumors (1–3). The importance of the preoperative differentiation of these subtypes has been gradually recognized due to their differences in lifestyle and genetic risk factors, patterns of spread, responses to chemotherapy, and prognoses. Borderline epithelial ovarian tumor (BEOT) with low malignant potential constitute a special histological type of EOT. Due to the younger age of onset, fertility-sparing surgery is a very important topic for consideration in BEOT patients (4, 5). In addition, adjuvant chemotherapy or radiotherapy is not recommended even in patients with advanced BEOT (6, 7). Differing from BEOT, epithelial ovarian cancer (EOC) accounts for the highest tumor-related mortality among women diagnosed with gynecological malignancy (2, 8). The dualistic model further classifies EOC into type I and type II, referring to the pathways of tumorigenesis (9, 10). Type I EOC develops in a stepwise fashion from well-established precursor lesions and has a good prognosis but low responsiveness to standard treatments such as platinum chemotherapy and hormonal treatments due to *KRAS* and *BRAF* mutations and high expression levels of c-Fos (11, 12). In contrast, type II EOC tends to present in advanced stages and has poorer outcomes. The reliable early identification of these subtypes contributes to the rational choice of treatment strategies and prognosis prediction (13).

Magnetic resonance imaging (MRI) is now widely applied in assessment of adnexal masses. As a functional imaging technique, diffusion weighted imaging (DWI) and the corresponding apparent diffusion coefficient (ADC) maps hold promise as additional tools for differentiating between the benign and malignant conditions of a particular disease and monitoring the course of therapy (14, 15). Previous studies have shown great capability of DWI sequences with ADC map for categorizing ovarian tumors (16, 17). However, no consensus has been reached regarding ADC measurements for the characterization of ovarian tumors. Radiomics has recently emerged as a powerful approach for non-invasively capturing the inter-lesion heterogeneity that can be used to build an objective and accurate decision support systems for cancer at low cost (18, 19). Innumerable quantitative features extracted using high-dimensional data from DWI and ADC map could reflect the underlying pathophysiology of tissue (20, 21). Previous studies have demonstrated the usefulness of histograms analysis based on ADC for the differential diagnosis of ovarian cancer (22, 23). Compared to the first-order features, the higher-order statistical features in DWI and ADC maps could better describe the diffusion pattern and heterogeneous distribution of tumor tissues. The present investigation used imaging features based on DWI and ADC maps to quantitatively characterize the properties of complex adnexal masses with the goal of improving the capability of diagnosing subtypes of EOT and providing guidance for clinicians to design specialized treatment plans. The complex ovarian masses often present cystic-solid characteristics. Most radiomics studies in EOTs have delineated regions of interest (ROIs) that cover all voxels, including hemorrhagic, necrotic, and cystic areas within the tumor (24, 25). The cystic components were relatively homogeneous when compare with the solid components. Thus, the ADC differences of the solid components might be compromised by a larger proportion of cystic components in the whole tumor (26). Therefore, whole-tumor ROI analysis with a prior ROI focused on the solid components of the lesion was conducted.

In the present study, a radiomics signature based on DWI and ADC maps was developed preoperatively to noninvasively classify EOTs into subtypes. Moreover, a comprehensive nomogram that incorporated the radiomics signature and clinical characteristics was established for the preoperative subtype differentiation of EOTs.

## Material and methods

### Patients

A total of 146 patients with surgically confirmed BEOT or EOC who underwent preoperative magnetic resonance imaging (MRI) examination between March 2016 and January 2021 were included. The exclusion criteria were as follows: (1) patients who received preoperative treatment; (2) MRI performed more than 1 month before surgery; and (3) poor image quality or maximum diameter of lesions < 1 cm.

Clinical data, including age, menopausal status, and CA-125 level, were obtained from the medical records. Two radiologists (with 4 and 8 years of experience in MRI interpretation) without knowledge of the clinical and histologic information evaluated the MRI data, and discrepancies were resolved by consensus. Information on tumor configuration, pelvic fluid, and peritoneal involvement were obtained. Tumor configuration was characterized as mainly cystic, mixed cystic-solid, and mainly solid. The institutional ethics committee approved this retrospective study and the informed consents were waived.

### MRI acquisition and tumor segmentation

**Figure 1:**
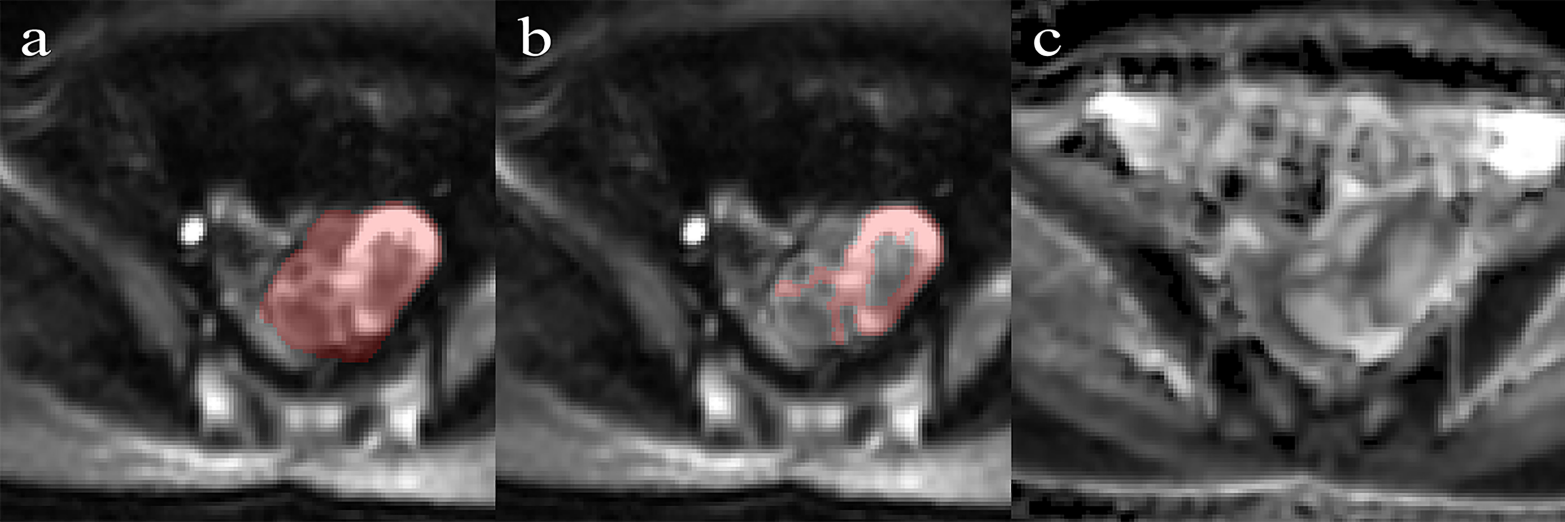
Performance of region of interest on the DWI (*b* = 1000 s/mm^2^) by referring to T2WI. **(A)** Whole-tumor ROIs are manually drawn along the edge of the tumor on the DWI. **(B)** Solid-tumor ROIs are drawn along the solid components of the tumor. **(C)** The corresponding ADC map.

MRI was performed using a 3.0T MR system (GE, Discovery 750W) with a phased-array coil. The scanning parameters of axial DWI were as follows: TE 70.5 msec; TR 4000 ms; FOV 34 cm; slice thickness (mm)/gap (mm) 5/1; flip angle 90; acquisition matrix 128 × 128; and *b* value 0 and 1000 s/mm^2^. The DWI sequence images were transferred to the workstation, and ADC maps were automatically calculated by a commercially available software package (Functool, GE Medical Systems). More details of MR image acquisition are provided in **Appendices A**. ROIs were manually segmented along the lesion on the largest slice using ITK-SNAP software (version 3.8.0, www.itksnap.org). Two different ROIs were positioned on the slice (**Figure 1**): (a) an ROI encompassing the whole tumor area; and (b) an ROI encompassing the solid part of the tumor area while avoiding hemorrhagic, necrotic, or cystic regions on the axial DWI images (*b* value of 1000 s/mm^2^) by referring to the T2-weighted images. ROIs were copied to the ADC map automatically.

### Feature extraction and selection

Before radiomics feature extraction, several preprocessing techniques were applied to standardized images to improve texture recognition. Radiomics features were extracted from *ROI_whole_* and *ROI_solid_* on DWI and ADC maps, respectively, using PyRadiomics (27). For each ROI, 9 shape features, 18 histogram features, and 75 texture features (24 gray level co-occurrence matrix (GLCM) features, 16 gray level run-length matrix (GLRLM) features, 16 gray level size-zone matrix (GLSZM) features, 14 gray level dependence matrix (GLDM) features, and 5 neighborhood gray tone difference matrix (NGTDM) features were calculated. Therefore, a total of 390 features were extracted for each lesion from both DWI and ADC maps. Detailed descriptions of the image preprocessing and feature extraction processes are provided in Appendices B.

The interobserver reproducibility was initially analyzed using 30 randomly chosen images for ROI segmentation. The Dice coefficient and Hausdorff distance were applied to estimate the similarity of ROIs. The features selection procedure included 4 steps. First, the radiomics features with poor reproducibility were removed. Second, univariate analysis was performed to select important features by using the Wilcoxon rank-sum test with a *P* value less than 0.05; third, the most significant predictive features were selected by using the least absolute shrinkage and selection operator (LASSO) logistic regression algorithm. Fourth, in multivariate logistic regression, backward stepwise selection was applied using a likelihood ratio test with Akaike’s information criterion as the stopping rule.

### Model construction

The shape features, first-order, and high-order image features from DWI and ADC maps were selected, and their performance in the discrimination of EOTs was evaluated separately. Then, all the important radiomics features were included in stepwise multivariate logistic regression analysis to construct a radiomics signature. For each patient, a score named as the radscore, was calculated Model construction was followed by 3-fold cross-validation repeated 50 times. A clinical model based on clinical characteristics and a nomogram with the Rad-score and clinical risk characteristics were also established.

**Figure 2:**
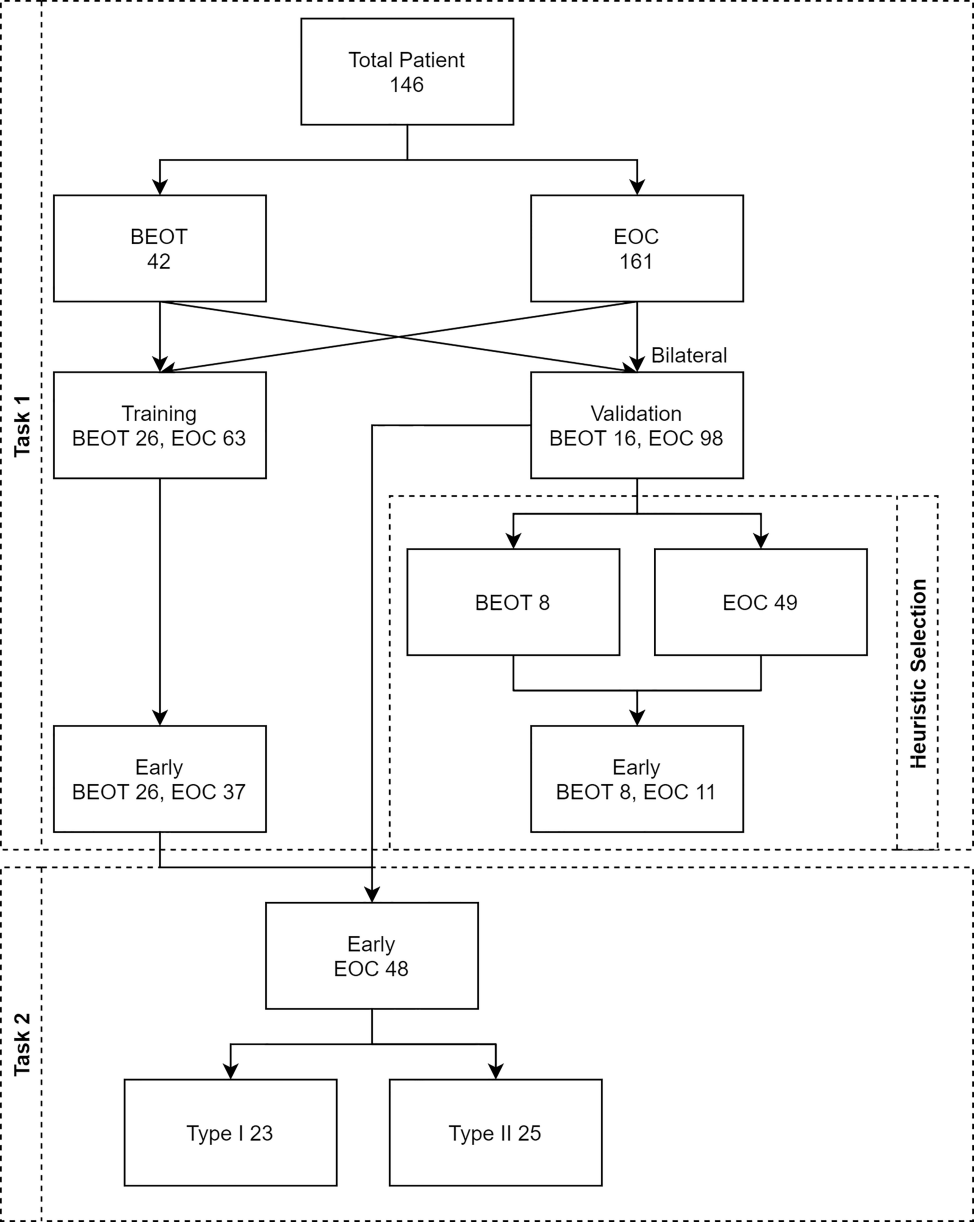
Flow diagram illustrating the two classification tasks. Task 1 was established for distinguishing BEOT from EOC, and task 2 was established from early-stage type I from II EOC. A total of 146 patients with 203 epithelial ovarian tumors, including 34 patients with 46 BEOT and 112 patients with 161 EOC.

Two classification tasks were assessed and shown in **Figure 2**: 1) BEOT vs. EOC and 2) early-stage (I-II) type I vs. type II EOC. For the classification of BEOT and EOC, patients with a single lesion were included in the training cohort, and patients with bilateral lesions were included in the validation cohort. Three distinct strategies were applied for the validation cohort: 1) both tumors were taken as independent samples; 2) the more complex tumor based on image features identified by radiologists; and 3) computer-assisted screening was established to select the tumor with maximum probability for final consideration. In addition, the diagnostic performance in differentiating between BEOT and early-stage EOC was also assessed. For the classification of early-stage type I and type II EOCs, all single and bilateral tumors were used as independent samples because of the small sample size.

### Statistical analysis

The Mann-Whitney *U* test and Pearson’s chi-square test were applied to assess the differences in the clinical characteristics of the patients. The interobserver reproducibility was evaluated using intraclass correlation coefficients (ICCs), and an ICC value less than 0.75 was considered poor reproducibility. The area under the receiver operating characteristic (ROC) curve (AUC), accuracy, sensitivity, and specificity were employed to quantitatively measure the discrimination capability of all the models. The 95% CI are computed with 2000 stratified bootstrap replicates. The Hosmer–Lemeshow test was performed to quantitatively assess the calibration and agreement between the predicted and observed results of all models. The DeLong test was used to determine whether significant differences existed in terms of the AUC values among these models. Decision curve analysis (DCA) was applied to compare the net benefits of the different models at different threshold probabilities. The net reclassification index (NRI) was applied to measure the prediction increment of the radiomics signature (28). All statistical analyses were performed in R software (version 3.6.3; https://www.rproject.org). Two-tailed *p* values less than 0.05 were considered statistically significant.

## Results

### Patients characteristics

The patients’ clinical characteristics are presented in **Table 1**. In terms of distinguishing BEOT from EOC, patient age, menopausal status, CA-125 level, bilaterality, MR-reported pelvic fluid, tumor configuration, and peritoneal involvement were significantly different (all *P* < 0.05), whereas no significant differences were detected in the bilaterality (*P* > 0.05). In terms of distinguishing early-stage type I and type II EOCs, patient age, CA-125 level, MR-reported pelvic fluid, tumor configuration and peritoneal involvement were significantly different (all *P* < 0.05), whereas no significant differences were detected in the menopausal status of the patients (*P* > 0.05). Then, the clinical model and radiomics nomogram were constructed by using the significant clinical characteristics.

**Table 1:** Patients characteristics.

	BEOT (n=34)	EOC (n=112)	*P* value	Type I EOC (n=30)	Type II EOC (n=82)	*P* value
Age (year)	38.5 (27.0;47.0)	57.0 (50.0;64.0)	< 0.001	55.0 (47.0;60.0)	58.0 (51.0;66.8)	0.047
CA125 level	18.1 (8.46;40.8)	54.2 (20.2;204)	0.001	36.0 (16.3;76.4)	94.2 (21.5;250)	0.018
Menopausal (%)	7/34 (20.6%)	74/112 (66.1%)	< 0.001	18/30 (60.0%)	56/82 (68.3%)	0.551
Early-stage (%)	34/34 (100%)	48/112 (42.9%)	–	23/30 (76.7%)	25/82(30.5%)	< 0.001
Bilaterality (%)	8/34 (23.5%)	49/112 (43.8%)	0.055	4/30 (13.3%)	45/82 (54.9%)	< 0.001
MRI reported fluid (%)	8/34 (23.5%)	68/112 (60.7%)	< 0.001	11/30 (36.7%)	57/82 (69.5%)	0.003
MRI reported peritoneal metastasis (%)	2/34(5.9%)	45/112 (40.2%)	< 0.001	3/30 (10.0%)	42/82 (51.2%)	< 0.001
MRI reported tumor configuration (%)						
Mainly cystic	28/42 (66.7%)	52/161 (32.3%)		16/34 (47.1%)	36/127 (28.3%)	
Mixed cystic-solid	5/42 (11.9%)	47/161 (29.2%)	0.001	13/34 (38.2%)	34/127 (26.8%)	0.002
Mainly solid	9/42 (21.4%)	62/161 (38.5%)		5/34 (14.7%)	57/127 (44.9%)	

BEOT, borderline epithelial ovarian tumor; EOC, epithelial ovarian cancer.

### Feature selection and performance of the radiomics signature for distinguishing BEOT from EOC

The mean Dice coefficient and Hausdorff distance were 0.810 and 6.750 for DWI and 0.935 and 9.149 for ADC, respectively. Detailed information regarding the ICCs is shown in Appendices C. Among 390 radiomics features, 2 shape features, 4 features from DWI, and 3 features from ADC were selected using the multivariate LASSO method, and the prediction performance outcomes of the shape features, DWI features, and ADC map features were separately evaluated, and the result are shown in Appendices D. Then, a stepwise multivariable logistic regression algorithm was applied to build the radiomics signature by using the selected features. Finally, 3 features, namely, ADC_solid_glszm_LowGrayLevelZoneEmphasis, ADC_solid_glcm_lmc1, and ADC_solid_Skewness, were included in the radiomics signature. The distribution of features is shown in **Appendices E**. Rad-score, menopausal status, and CA-125 level were identified as independent factors for discriminating between BEOT and EOC.

**Figure 3:**
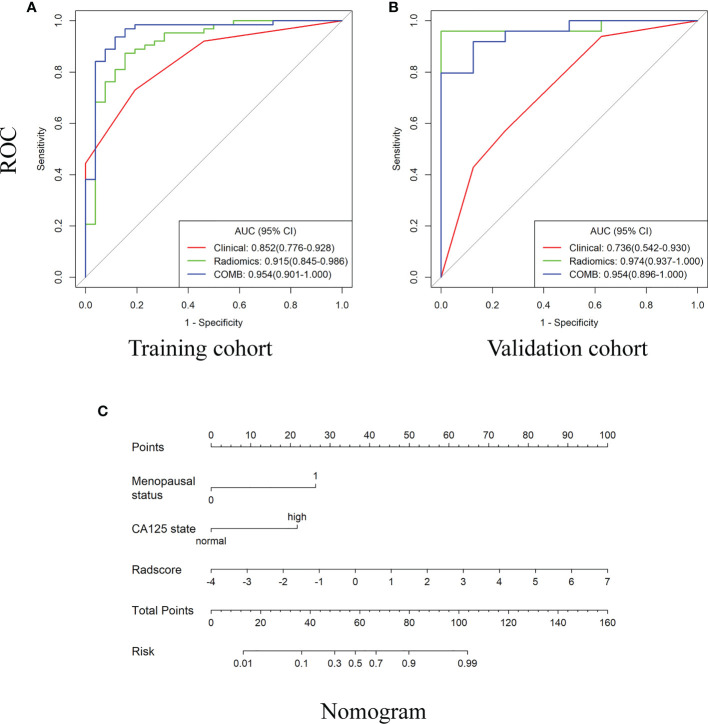
The receiver operating characteristic (ROC) curves and the nomogram for preoperatively distinguishing borderline epithelial ovarian tumor from epithelial ovarian cancer. **(A–B)** The ROC curves of clinical, radiomics and nomogram in training and validations cohorts. **(C)** The DWI-based radiomics nomogram.

**Figure 4:**
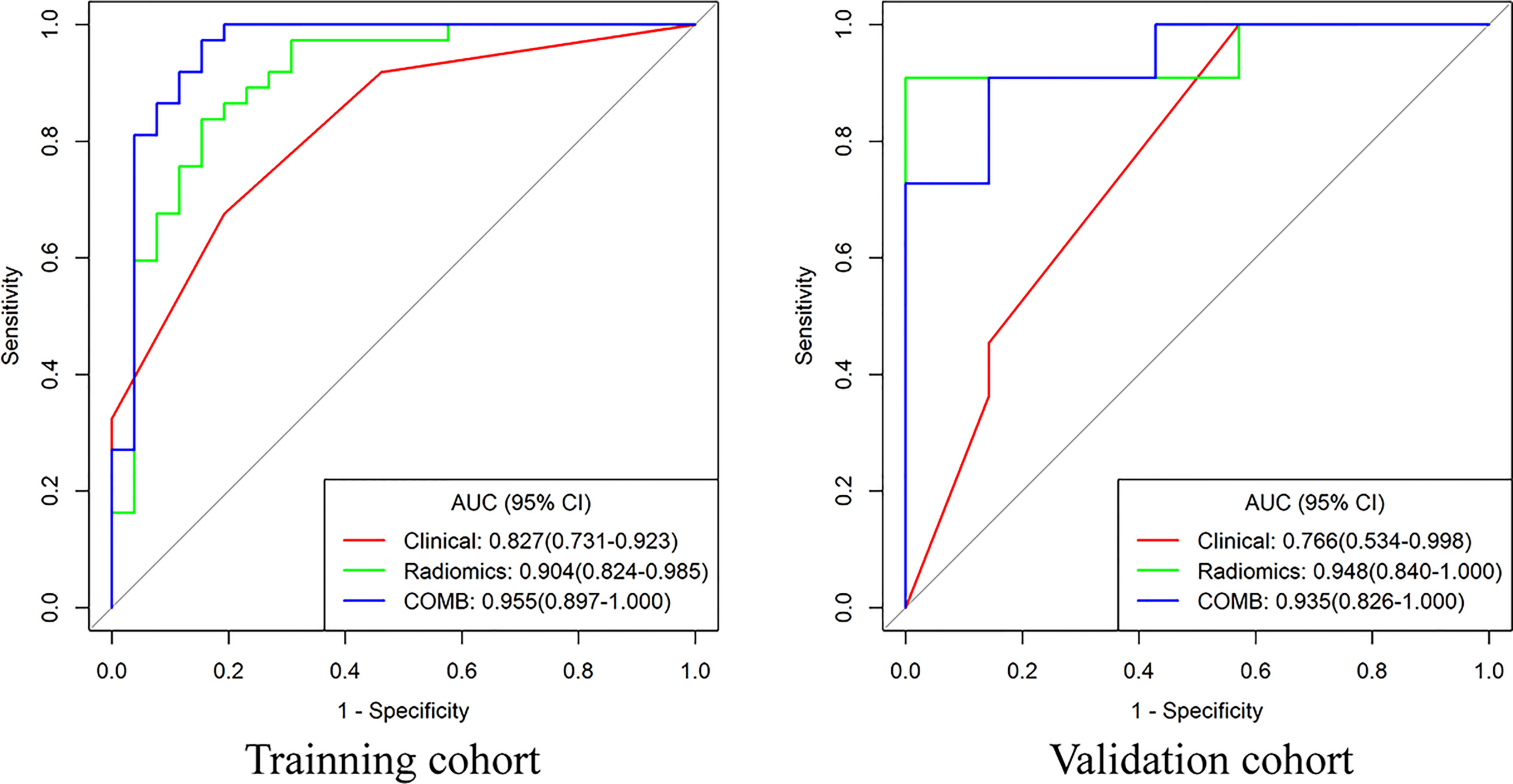
The receiver operating characteristic curves of clinical model, radiomics and nomogram for distinguishing BEOT from early-stage EOC.

Compared with the clinical model, the radiomics signature and nomogram showed better performance for distinguishing BEOT from EOC in the training cohort (0.915 vs. 0.852, *P* = 0.21; 0.954 vs. 0.852, *P* = 0.01) and in the validation cohort (0.974 vs. 0.736, *P* = 0.01; 0.954 vs. 0.736, *P* = 0.004) by tumor. The accuracy, sensitivity, specificity, positive predictive value, negative predictive value and their 95% CI were shown in **Table 2**. For the validation cohort by patients, the maximum probability selection method achieved a higher diagnostic performance than the two methods mentioned above (more detailed information is shown in Appendices F). NRI with value of 0.5791 (95% CI: 0.2162 - 0.942, *P* = 0.00176) in comparing between maximum probability selection method and radiologist selection method. The diagnostic performance for distinguishing BEOT from EOC in the training and validation cohorts was presented by ROC curves. DCA showed that using either the radiomics signature or nomogram adds more benefit than using the clinical model. Good calibration was observed, and the Hosmer-Lemeshow test showed the goodness-of-fit of the radiomics signature (*P* = 0.074 and 0.663) and nomogram (*P* = 0.175 and 0.207) (more detailed information regarding the calibration curves and DCA are shown in Appendices G). **Figure 3** illustrates the ROC curve and the nomogram for preoperatively distinguishing BEOT from EOC. In addition, the predictive performance for distinguishing BEOT from early-stage EOC was also determined. As shown in **Figure 4**, the radiomics signature and nomogram showed better performance than the clinical model in distinguishing BEOT from early-stage EOC in the training cohort (AUC: 0.904 vs. 0827, *P* = 0.23; AUC: 0.955 vs. 0.827, *P* = 0.013) and in the validation cohort (AUC: 0.948 vs. 0.766, *P* = 0.15; AUC: 0.936 vs. 0.766, *P* = 0.08). More detailed information regarding the results is provided in Appendices H.

**Table 2:** Diagnostic performance of clinical model, radiomics, and nomogram in differentiating BEOT from EOC.

	Training cohort			Validation cohort		
	Clinical model	Radiomics	Nomogram	Clinical model	Radiomics	Nomogram
AUC (95% CI)	0.852(0.776-0.928)	0.915(0.845-0.986)	0.954(0.901-1.000)	0.736(0.603-0.869)	0.974(0.937-1.000)	0.954(0.896-1.000)
Accuracy (95% CI)	0.753(0.650-0.838)	0.865(0.776-0.928)	0.921(0.845-0.968)	0.596(0.501-0.687)	0.930(0.830-0.981)	0.842(0.721-0.925)
Sensitivity (95% CI)	0.730(0.546-0.864)	0.873(0.698-0.968)	0.937(0.738-1.000)	0.571(0.342-0.778)	0.918(0.898-1.000)	0.837(0.694-1.000)
Specificity (95% CI)	0.808(0.630-0.923)	0.846(0.615-0.962)	0.885(0.731-1.000)	0.750(0.524-0.938)	1.000(0.497-1.000)	0.875(0.625-1.000)
PPV (95% CI)	0.902(0.873-0.916)	0.932(0.917-0.938)	0.952(0.939-0.955)	0.933(0.894-0.950)	1.000(1.000-1.000)	0.976(0.971-0.980)
NPV (95% CI)	0.553(0.491-0.585)	0.733(0.667-0.758)	0.852(0.826-0.867)	0.222(0.167-0.263)	0.667(0.498-0.667)	0.467(0.385-0.500)

BEOT, borderline epithelial ovarian tumor; EOC, epithelial ovarian cancer; AUC, area under curve; PPV, positive predictive value; NPV, negative predictive value; 95% CI, 95% confidence interval.

### Feature selection and performance of the radiomics signature for distinguishing early-stage type I from II EOC

**Figure 5:**
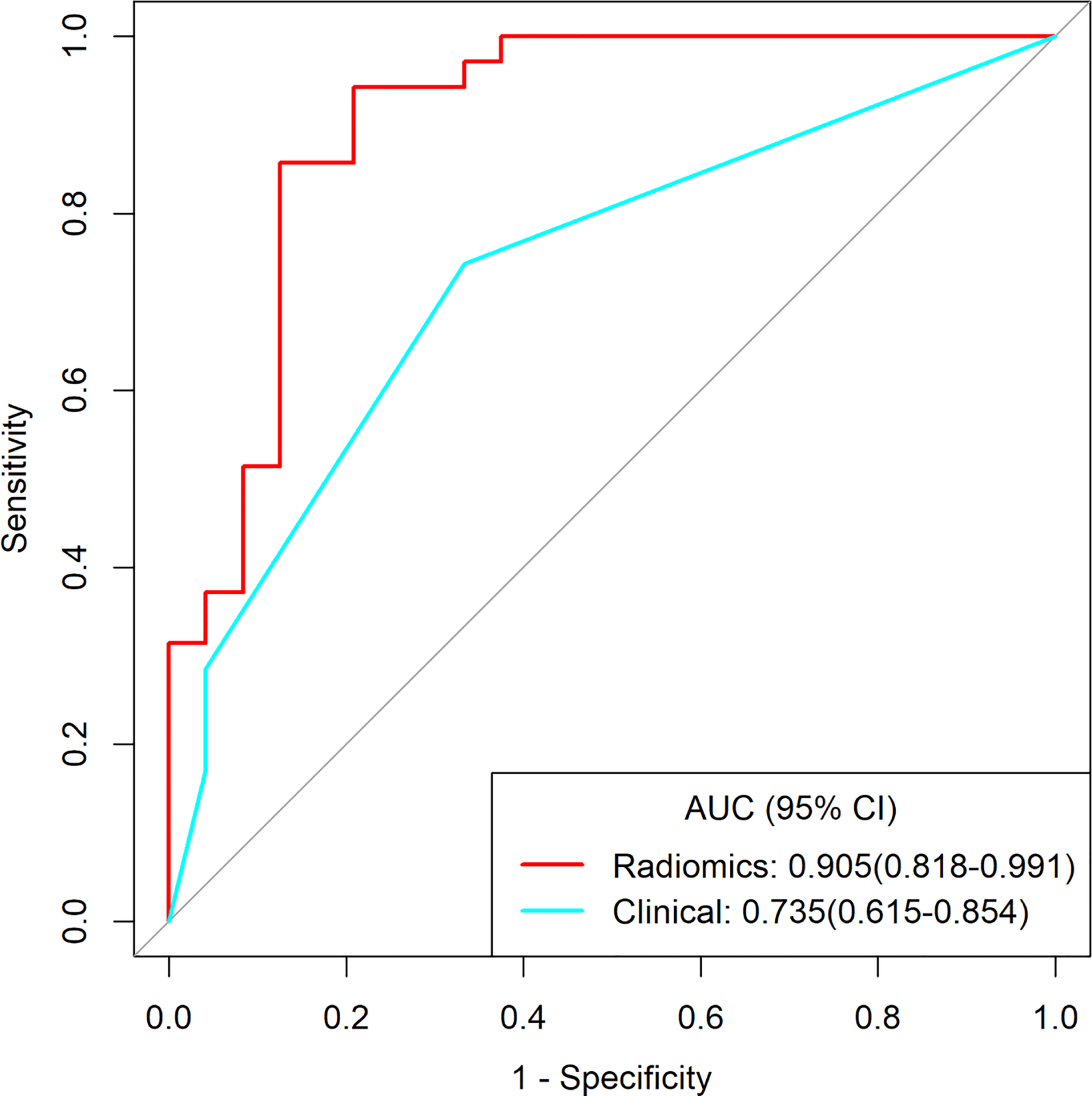
The receiver operating characteristic curves of clinical model and radiomics for distinguishing early-stage type I and type II EOC.

Among 390 radiomics features, 10 potential features were selected using the LASSO method. The selected features were then applied to build the radiomics signature by using a stepwise multivariable logistic regression algorithm. Finally, 4 radiomics features (whole_MinorAxisLength, DWI_whole_glrlm_GrayLevelVariance, DWI_solid_gldm_ LargeDependenceHighGrayLevelEmphasis, and ADC_whole_glrlm_ ShortRunLowGrayLevelEmphasis) were identified for inclusion in the radiomics signature. The distribution of features is shown in **Appendices I**. **Table 3** summarizes the diagnostic performance of the clinical model, radiomics signature, and nomogram. The radiomics signature showed favorable discrimination, with an AUC of 0.905 with accuracy, sensitivity, and specificity of 88.1%, 94.3% and 79.2%, respectively. Rad-score, pelvic fluid, and tumor configuration were identified as independent factors for discriminating between early-stage type I and type II EOC through univariate and multivariable analyses. The radiomics signature performed significantly better than the clinical model (0.905 vs. 0.735, *P* = 0.007) in distinguishing early-stage type I EOC from type II EOC. The diagnostic efficacy of the nomogram was the same as that of the radiomics model with a NRI of -0.1591 (95% CI: -1.0516 - 0.7334, *P* = 0.7268). Good calibration was observed, and the Hosmer-Lemeshow test showed the goodness-of-fit of the data (*P* = 0.062). DCA showed that using the radiomics signature adds more benefit than using the clinical model. More detailed information regarding the calibration curves and DCA are shown in Appendices J. **Figure 5** shows the ROC for preoperatively classifying early-stage type I and type II EOCs.

**Table 3:** Diagnostic performance of clinical model, radiomics, and nomogram in classification between early-stage type I and II EOC.

	AUC (95% CI)	Accuracy (95% CI)	Sensitivity (95% CI)	Specificity (95% CI)	PPV (95% CI)	NPV (95% CI)
Clinical model	0.735(0.615-0.854)	0.712(0.579-0.822)	0.743(0.486-0.873)	0.667(0.375-0.834)	0.765(0.680-0.793)	0.640(0.500-0.690)
Radiomics	0.905(0.818-0.991)	0.881(0.771-0.951)	0.943(0.457-1.000)	0.792(0.542-0.958)	0.868(0.762-0.875)	0.905(0.867-0.920)
Nomogram	0.905(0.818-0.991)	0.881(0.771-0.951)	0.943(0.485-1.000)	0.792(0.500-0.958)	0.868(0.772-0.875)	0.905(0.857-0.920)

EOC, epithelial ovarian cancer; AUC, area under curve; PPV, positive predictive value; NPV, negative predictive value; 95% CI, 95% confidence interval.

## Discussion

EOTs are a group of tumors consisting of dissimilar cell types with different biological behaviors. Subtype differentiation is beneficial for the individualized treatment of EOTs because of the different disease courses. BEOT is characterized by mild nuclear atypia and a lack of stromal invasion, whereas EOC is characterized by high cellularity and abundant stromal invasion. According to the concept of radiomics, these differences could be reflected by quantitative analysis and radiomics methods. Our results demonstrated that the radiomics signature and the nomogram showed higher performance than the clinical model in differentiating BEOT and EOC and in classifying type I and type II EOCs. In addition, the maximum probability selection method achieved excellent diagnostic performance for distinguishing BEOT from EOC. Therefore, radiomics signatures derived from DWI and ADC maps may be powerful noninvasive imaging biomarkers for the subtype differentiation of EOTs.

Morphological changes in EOT can be observed by conventional ultrasound and MRI examination. However, it remains a challenge for differentiation diagnosis of EOT due to morphological complexity and overlap.The Assessment of Different NEoplasias in the adneXa model and Ovarian-Adnexal Reporting and Data System based on ultrasound appearances achieved good performance for the discrimination between benign and malignant adnexal tumor, but depending on an experienced examiner and high-end ultrasound equipment (29). The reproducible and noninvasive nature of radiomics provides clinician with a favorable approach to predict clinicopathological variables. Theoretically, radiomics features morphological and features decode subtype of EOTs differently. Several radiomics studies based on DWI have been established for ovarian cancer classification and histological grade evaluation. Mimura et al. (28) found that the 10th percentile of ADC values had the highest AUC for differentiating borderline ovarian tumors from malignant ovarian tumors. Li et al. (22) studied the potential of histogram features in the ADC map for grading serous ovarian carcinoma. Their results showed that all ADC histogram features except kurtosis are effective in distinguishing high-grade serous ovarian carcinoma from low-grade serous ovarian carcinoma. In contrast to previous studies that focused on histogram features, we extracted a large number of quantitative and minable imaging features to find more valuable information related to the subtype differentiation of EOTs. Our results showed that the features of Skewness, GLCM_lmc1 and GLSZM_LowGrayLevelZoneEmphasis were the key features for categorizing EOTs. The skewness features revealed that malignant EOT exhibited greater asymmetry with respect to value distributions about the mean as compared with borderline EOT. The high-order features of GLCM and GLSZM could quantify the spatial relationships and interactions between pixel intensities to capture the distinctive intratumour heterogeneity and subtle alterations in subtypes of EOTs. Our results demonstrated that radiomics presents a clinically applicable and cost-effective decision-making tool for personalized medicine in EOTs. The radiomics signature and nomogram showed better performance than the clinical model in discriminating BEOT from EOC, indicating that radiomics may help improve the diagnostic accuracy before invasive procedures. For patients with bilateral tumors, maximum probability selection was established and achieved excellent diagnostic performance for distinguishing BEOT from EOC. It is worth noting that the radiomics signature performed better than the nomogram, but without a significant difference. This discrepancy, however, may be due to the instability of clinical characteristics. In clinical practice, it is more difficult to distinguish between BEOT and early-stage EOC with limited tumor spread because advanced EOC tends to show more aggressive characteristics, such as peritoneal involvement, distant metastases, and ascites. Therefore, a subtask of distinguishing between BEOT and early-stage EOC was also performed and achieved a high overall classification performance, with an AUC value of 0.904 in the training cohort.

A dualistic model classifies EOC into two broad categories designated type I and type II based on the pathogenesis and origin. Zhang et al. (25) reported that radiomics features extracted from MRI yielded excellent performance in classifying type I and type II ovarian cancers. However, only intensity information in the ADC map was analyzed. Jian et al. (24) constructed a multiparametric MRI model for differentiating between type I and type II EOC. Although some algorithms have been proposed for the classification of type I and type II EOC, clinical characteristics were not incorporated. In this study, a radiomics signature achieved better performance than the clinical model in discriminating early-stage type I EOC from type II EOC. However, the nomogram comprising radiomics features and clinical characteristics showed same diagnostic efficacy as the radiomics with NRI value of -0.1591. These results indicate that clinical factors have little effect on the nomogram for distinguishing the early-stage type I EOC from type II EOC and the radiomics features could be an effective quantitative approach to categorize EOTs. Radiomics provides a more objective and accurate way for gynecologists to develop a customized process to maximize the success of preventive and therapeutic interventions with minimum side effects in patients with EOT.

In addition, the ROI methods have varied between ovarian radiomics studies and have not achieved consensus. The accuracy of EOT classification often depends on the feature expression of the ROI. As a complex mass comprising solid and cystic components, the features from the whole tumor or the solid components alone may not be sufficiently accurate to distinguish the subtypes of EOTs. In this study, whole-tumor ROI analysis with a prior ROI focused on the solid components of ovarian lesions was performed. The present results demonstrate that ROIs reflect the different characteristics of tumors, and their combination can more comprehensively reflect the internal heterogeneity of ovarian tumors.

Several limitations should be noted. First, for a radiomics study, the sample size of a single center, such as ours, is arguably somewhat small. A multicenter, large-scale trial should be performed to validate our preliminary results. Second, lesion segmentation was manually outlined on a single slice. Undoubtedly, volumetric tumor delineation could provide a more comprehensive evaluation of the underlying spatial heterogeneity, but the analysis is time-consuming for clinical application. A two-dimensional analysis may be more highly recommended for clinical application (30). More studies are warranted to explore the optimal tumor segmentation approach for clinical application. Finally, the ADC values used in this study were derived from a monoexponential diffusion model, and features of other parameter maps derived from DWI images using the biexponential or stretched-exponential diffusion mode will be considered in our future work for ovarian tumors.

In this present study, imaging features were extracted from DWI scans of ovarian tumors. The results demonstrated that the subtype of EOTs could be predicted based on imaging features from DWI and the nomogram. Future studies with larger sample sizes and more radiomic features should be conducted to refine our findings.

## Data availability statement

The original contributions presented in the study are included in the article/**Supplementary Material**. Further inquiries can be directed to the corresponding authors.

## Ethics statement

The studies involving human participants were reviewed and approved by The institutional ethics committee of the Shanxi Medical University Second Hospital. Written informed consent for participation was not required for this study in accordance with the national legislation and the institutional requirements.

## Author contributions

YX: methodology, resources, and data curation. H-JL: methodology, conceptualization, and investigation. L-MG: conceptualization and supervision. YX and H-JL: writing - original draft and data curation. JR: resources and data curation. XS and JN: project administration, supervision, and funding acquisition. All authors contributed to the article and approved the submitted version.

## Funding

This work is supported by the Applied Basic Research Programs of Shanxi Province (grant number 20210302123290).

## Conflict of interest

The author, JR, was employed by GE Healthcare.

The remaining authors declare that the research was conducted in the absence of any commercial or financial relationships that could be construed as a potential conflict of interest.

## Publisher’s note

All claims expressed in this article are solely those of the authors and do not necessarily represent those of their affiliated organizations, or those of the publisher, the editors and the reviewers. Any product that may be evaluated in this article, or claim that may be made by its manufacturer, is not guaranteed or endorsed by the publisher.
