# Supplementary material for: Diffusion-weighted imaging-based radiomics in epithelial ovarian tumors: Assessment of histologic subtype
Source: Front Oncol. 2022 Dec 5;12:978123. doi: 10.3389/fonc.2022.978123 (PMC9762272; doi:10.3389/fonc.2022.978123)
Supplement: Supplementary file 1 [file DataSheet_1.docx]

**Supplemental Materials**

**A: Acquisition of parametric maps**

Quantitative maps were automatically generated with the implementation manufacturer’s software of Functool (GE Advantage 4.6 workstation). Apparent diffusion coefficient (ADC) map was derived from monoexponential model with two *b*-value of 0 and 1000 s/mm^2^. ADC values can be calculated using the following equation: S/S0=exp (−*b* × ADC), where S represents the degree of signal attenuation, S0 means signal intensity of the diffusion weighted image with no diffusion gradient applied, and *b* value means degree of diffusion weighting.

**B: Image pre-processing and features extraction process**

Prior to tumor segmentation, preprocessing techniques were applied to standardized images in order to improve texture recognition. First, we resampled the images using bilinear interpolation to a 1.5 mm x 1.5 mm x 1.5 mm/pixel for all images. Second, grayscale normalization is indispensable prior to radiomics feature calculation due to the different original grayscales of the DWI. Each image was first normalized to achieve a mean and unit variance of 0. And image pre-processing comprised gray-level discretization in 10 different bin widths. Then, Z-score standardization was performed on DWI images to remove values greater than three standard deviations. Last, remove error pixels from ADC diagram (pixel value less than 0).

Radiomics features were extracted from ROI_whole and ROI_solid on DWI and ADC maps respectively. For each ROI, 9 shape features, 18 histogram feature, and 93 texture features [24 gray-level cooccurrence matrix (GLCM) features, 16 gray-level run length matrix (GLRLM) features, 16 gray-level size zone matrix (GLSZM) features, 14 gray-level dependence matrix (GLDM) features, and 5 neighborhood gray-tone difference matrix (NGTDM) features] were derived. Therefore, a total of 390 features were extracted for each lesion from both DWI and ADC maps. Ultimately, 390 quantitative 2D radiomics features $390= 9x2+(18+24+16+16+14+5)\times2+(18+24+16+16+14+5)\times2$.

**C: ICC results of feature selection**

The mean Dice coefficient and Hausdorff distance were 0.942 and 13.26 for ROI_solid and 0.971 and 6.753 for ROI_whole, respectively, which demonstrating the manual drawing with high repeatability.

Features in which the variance was close to 0 and with low reproducibility (interobserver ICC < 0.75) were excluded. The number of DWI and ADC map features was reduced to 302 and 294, respectively.

**D: Performance of shape, DWI features, and ADC maps features**

| Table D1 Diagnostic performance of shape features, DWI features, and ADC map features in distinguishing BEOT from EOC | | | | | | |
| --- | --- | --- | --- | --- | --- | --- |
|  | Training cohort | | | Validation cohort | | |
|  | Shape | DWI | ADC | Shape | DWI | ADC |
| AUC | 0.793(0.693-0.893) | 0.883(0.811-0.954) | 0.915(0.845-0.986) | 0.753(0.648-0.858) | 0.732(0.587-0.878) | 0.895(0.808-0.983) |
| Accuracy | 0.787(0.687-0.866) | 0.831(0.737-0.902) | 0.865(0.776-0.928) | 0.789(0.703-0.860) | 0.737(0.646-0.815) | 0.877(0.803-0.931) |
| Sensitivity | 0.841(0.508-0.921) | 0.810(0.539-0.905) | 0.873(0.666-0.969) | 0.857(0.652-0.980) | 0.776(0.592-0.990) | 0.898(0.571-1.000) |
| Specificity | 0.654(0.268-0.808) | 0.885(0.577-1.000) | 0.846(0.615-0.962) | 0.375(0.125-0.625) | 0.500(0.311-0.750) | 0.750(0.561-0.938) |
| PPV | 0.855(0.780-0.866) | 0.944(0.919-0.950) | 0.932(0.913-0.938) | 0.894(0.865-0.906) | 0.905(0.879-0.924) | 0.957(0.933-0.961) |
| NPV | 0.630(0.411-0.677) | 0.657(0.556-0.684) | 0.733(0.667-0.758) | 0.300(0.125-0.417) | 0.267(0.184-0.353) | 0.545(0.473-0.600) |
| AUC: area under curve; PPV: positive predictive value; NPV: negative predictive value | | | | | | |

**
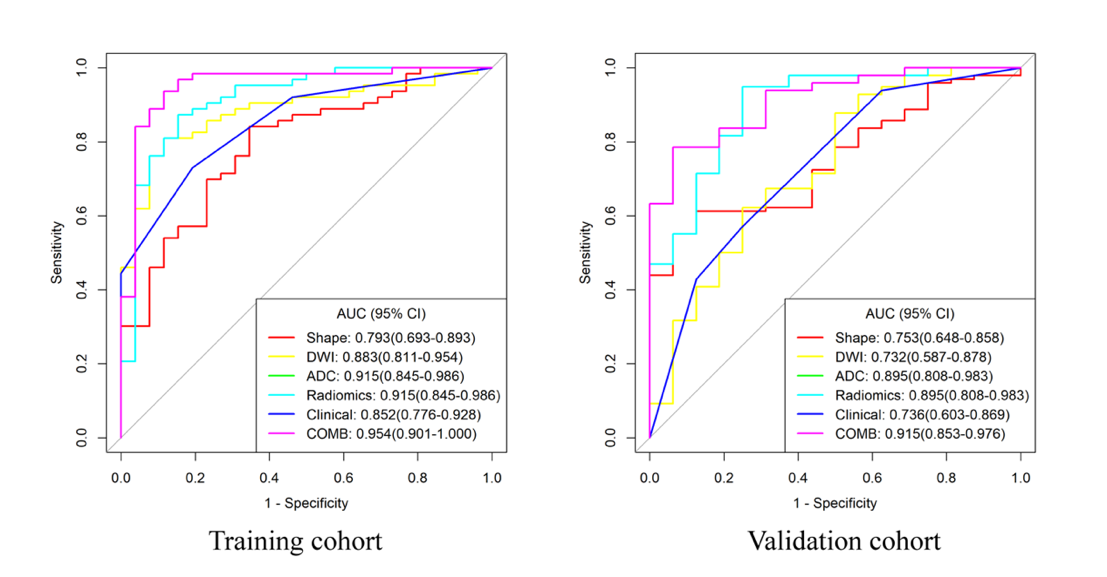
**

Figure D1. Diagnostic performance of each model in the training, and validation cohorts in classification between BEOT from EOC

**E: Distribution of each feature in distinguishing BEOT from EOC**

**
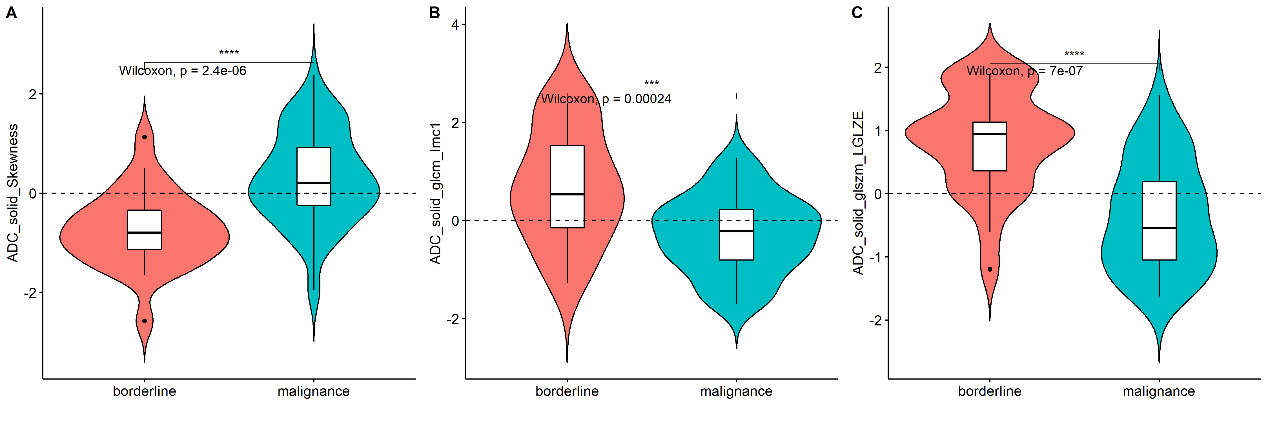
**

Figure E1. Distribution of ADC_solid_Skewness (A), ADC_solid_glcm_lmc1(B), and ADC_solid_glszm_LowGrayLevelZoneEmphasis (C) in distinguishing BEOT from EOC

**F: Diagnostic performance of each method in validation cohort**

| Table F1 Diagnostic performance of each method in validation cohort and validation cohort | | | | | | |
| --- | --- | --- | --- | --- | --- | --- |
|  | Both lesions selection | | Radiologist selection | | Heuristic selection | |
| Model | Radiomics | Nomogram | Radiomics | Nomogram | Radiomics | Nomogram |
| AUC | 0.895(0.808-0.983) | 0.915(0.853-0.976) | 0.929(0.836-1.000) | 0.916(0.824-1.000) | 0.974(0.937-1.000) | 0.954(0.896-1.000) |
| Accuracy | 0.877(0.803-0.931) | 0.842(0.762-0.904) | 0.877(0.763-0.949) | 0.842(0.721-0.925) | 0.930(0.830-0.981) | 0.842(0.721-0.925) |
| Sensitivity | 0.898(0.602-0.990) | 0.867(0.755-0.990) | 0.898(0.652-1.000) | 0.878(0.755-1.000) | 0.918(0.898-1.000) | 0.837(0.694-1.000) |
| Specificity | 0.750(0.500-0.938) | 0.688(0.500-0.938) | 0.750(0.500-1.000) | 0.625(0.375-1.000) | 1.000(0.497-1.000) | 0.875(0.625-1.000) |
| PPV | 0.957(0.936-0.960) | 0.944(0.937-0.951) | 0.957(0.941-0.961) | 0.935(0.925-0.942) | 1.000(1.000-1.000) | 0.976(0.971-0.980) |
| NPV | 0.545(0.444-0.600) | 0.458(0.381-0.536) | 0.545(0.444-0.615) | 0.455(0.333-0.571) | 0.667(0.498-0.667) | 0.467(0.385-0.500) |
| AUC: area under curve; PPV: positive predictive value; NPV: negative predictive value | | | | | | |

**G: Calibration curve in classification between BEOT and EOC**

**
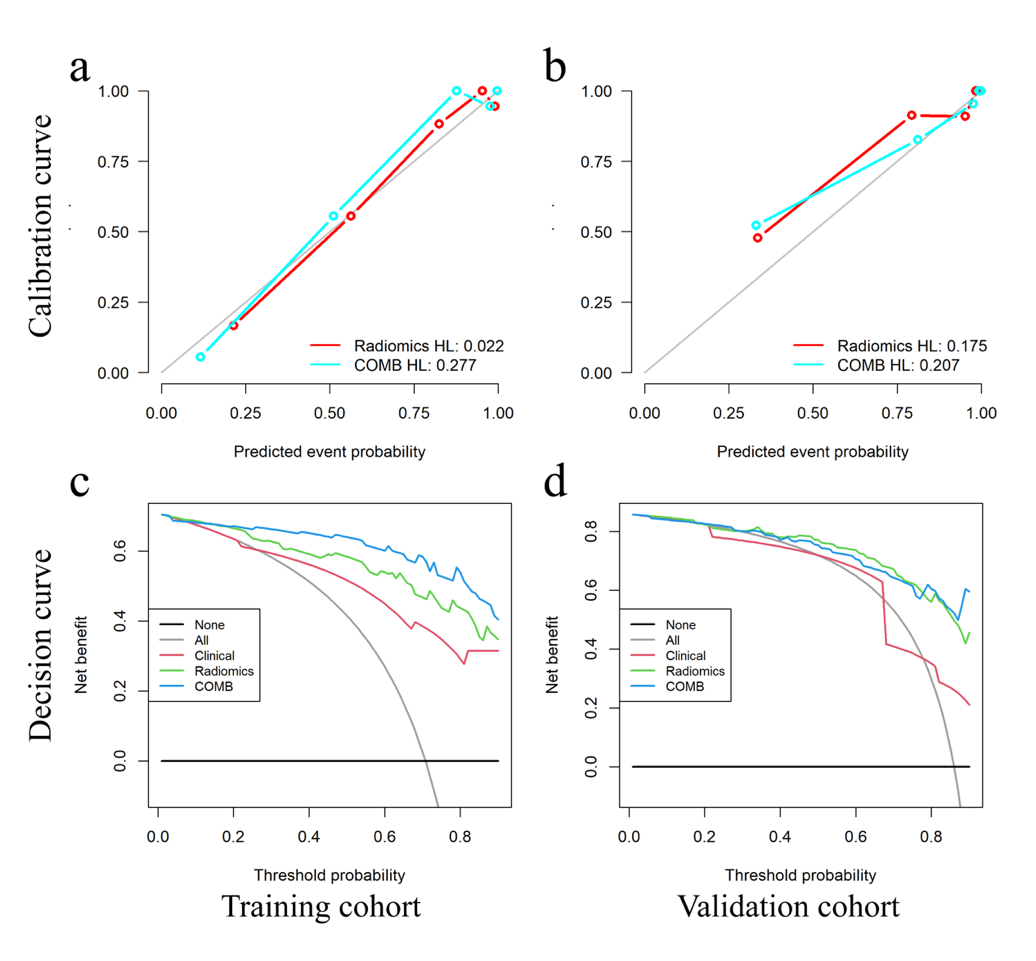
**

Figure G1. Calibration curve (a-b) and decision curve (c-d) in the training and validation cohorts in classification between BEOT and EOC

**H: Diagnostic performance of clinical model, radiomics, and nomogram in classification BEOT from early-stage EOC**

| Table H1. Diagnostic performance of clinical model, radiomics, and nomogram in classification BEOT from early-stage EOC | | | | | | |
| --- | --- | --- | --- | --- | --- | --- |
|  | Training cohort | | | Validation cohort | | |
| Model | Clinical model | Radiomics | Nomogram | Clinical model | Radiomics | Nomogram |
| AUC | 0.827(0.731-0.923) | 0.904(0.824-0.985) | 0.955(0.897-1.000) | 0.766(0.534-0.998) | 0.948(0.840-1.000) | 0.935(0.826-1.000) |
| Accuracy | 0.730(0.603-0.834) | 0.841(0.727-0.921) | 0.905(0.804-0.964) | 0.611(0.357-0.827) | 0.889(0.653-0.986) | 0.778(0.524-0.936) |
| Sensitivity | 0.676(0.456-0.865) | 0.838(0.541-0.973) | 0.919(0.757-1.000) | 0.455(0.091-1.000) | 0.818(0.727-1.000) | 0.727(0.545-1.000) |
| Specificity | 0.808(0.597-0.940) | 0.846(0.577-0.962) | 0.885(0.731-1.000) | 0.857(0.606-1.000) | 1.000(0.429-1.000) | 0.857(0.714-1.000) |
| PPV | 0.833(0.771-0.865) | 0.886(0.833-0.900) | 0.919(0.903-0.925) | 0.833(0.500-0.917) | 1.000(1.000-1.000) | 0.889(0.857-0.917) |
| NPV | 0.636(0.564-0.671) | 0.786(0.714-0.806) | 0.885(0.864-0.897) | 0.500(0.414-0.538) | 0.778(0.600-0.778) | 0.667(0.625-0.700) |
| AUC: area under curve; PPV: positive predictive value; NPV: negative predictive value | | | | | | |

**I: Distribution of each feature in distinguishing early-stage type I from type II EOC**


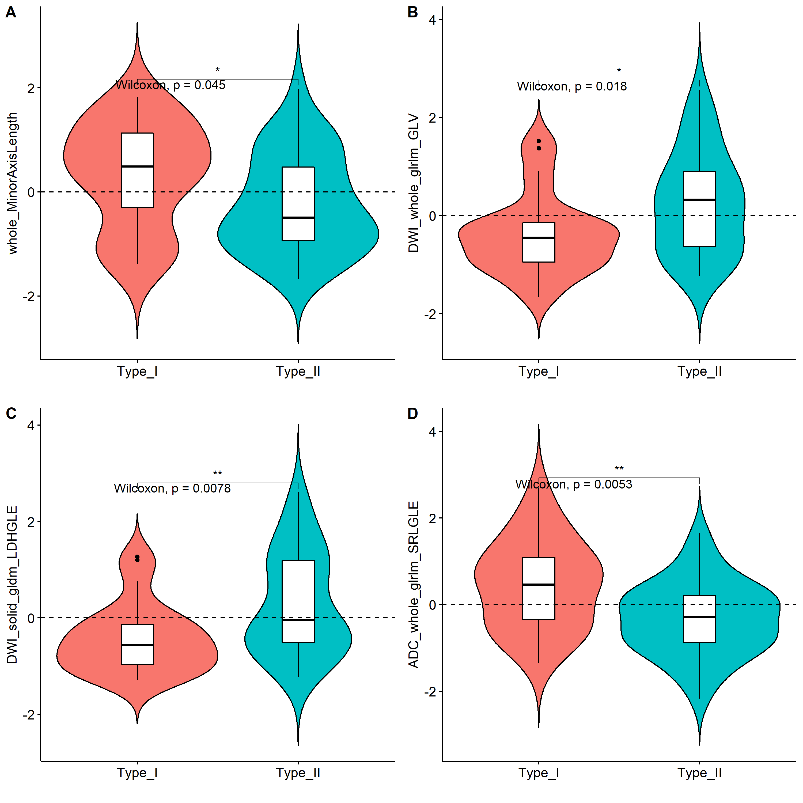


Figure I1. Distribution of whole_MinorAxisLength (A), DWI_whole_glrlm_GrayLevelVariance (B), DWI_solid_gldm_LargeDependenceHighGrayLevelEmphasis (C), and ADC_whole_glrlm_ShortRunLowGrayLevelEmphasis (D) in distinguishing early-stage type I from type II EOC

**J: Calibration curve in classification between early-stage type I and type II EOC**


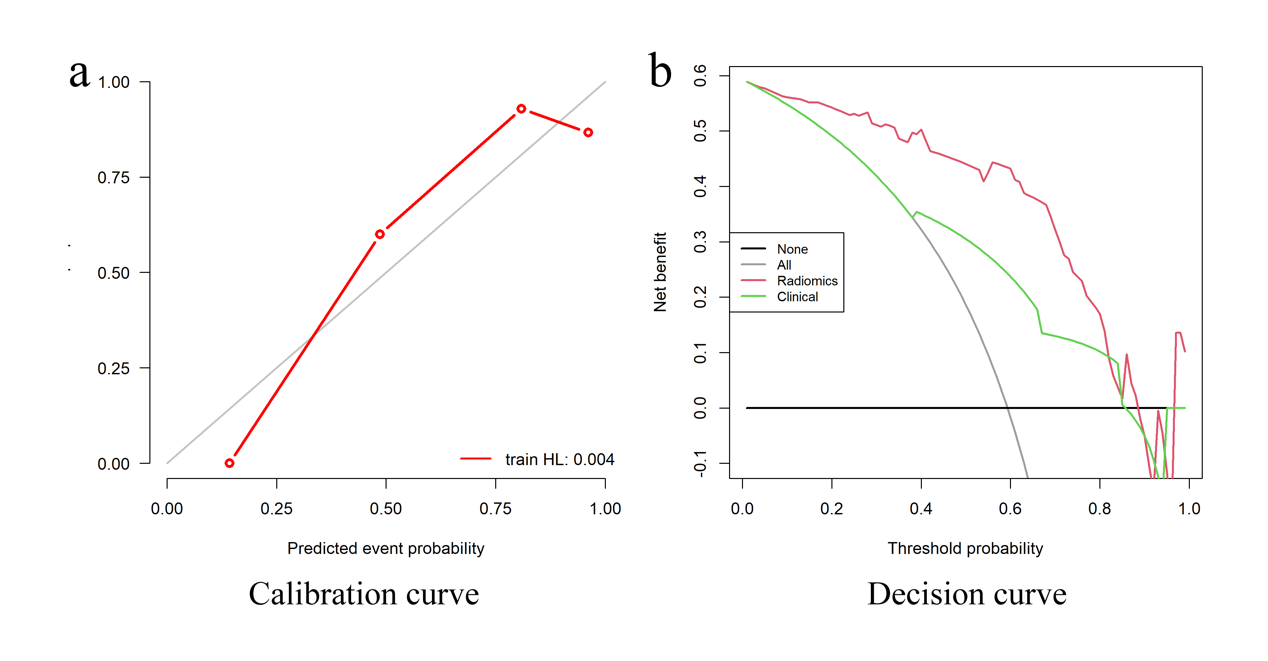


Figure J1. Calibration curve (a) and decision curve (b) in classification between early-stage type I and type II EOC
